# Leveraging diverse cell-death related signature predicts the prognosis and immunotherapy response in renal clear cell carcinoma

**DOI:** 10.3389/fimmu.2023.1293729

**Published:** 2023-12-11

**Authors:** Zhengqi Wu, Mingyue Jin, Peng Xin, Hao Zhang

**Affiliations:** ^1^ Department of Urology, The First Hospital of China Medical University, Shenyang, Liaoning, China; ^2^ Department of Endocrinology, Shenzhen University General Hospital, Shenzhen, Guangdong, China

**Keywords:** clear cell renal cell carcinoma, programmed cell death, prognosis, immunotherapy efficacy, IDUA

## Abstract

**Background:**

Modulation of programmed cell death in tumor cells alters the tumor microenvironment and the influx of tumor-infiltrating lymphocytes, and the combination of its inducers and immune checkpoint inhibitors plays a synergistic role in enhancing antitumor effects.

**Methods:**

We downloaded the data of clear cell renal cell carcinoma samples from The Cancer Genome Atlas and used a machine learning approach to build a new programmed cell death index (PCDI) through 13 programmed cell death-related genes. Based on PCDI, clinical features, tumor immune microenvironment, chemotherapy response and immunotherapy response were systematically analyzed.

**Results:**

PCDI consists of eight programmed cell death-related genes (TBX3, BID, TCIRG1, IDUA, KDR, PYCARD, IFNG and LRRK2). PCDI is a reliable predictor of survival in clear cell renal cell carcinoma patients and has been validated in multiple external datasets. We found that the high PCDI group showed higher levels of immune cell infiltration and better response to immunotherapy compared to the low PCDI group, and PCDI can also be used for prognostic prediction in a variety of cancers other than clear cell renal cell carcinoma. *In vitro* experiments demonstrated that knockdown of IDUA inhibited the proliferation and migration of clear cell renal cell carcinoma.

**Conclusions:**

The PCDI identified in this study provides valuable insights into the clinical management of clear cell renal cell carcinoma by accurately evaluating the prognosis of patients with clear cell renal carcinoma and identifying the patient population that would benefit from immunotherapy.

## Background

Clear cell renal cell carcinoma (ccRCC) is the main histologic subtype of renal cell carcinoma and also a common urologic malignancy (1). Despite substantial advances in immunotherapy and targeted therapies for ccRCC, patients with advanced disease still have high recurrence and mortality rates (2, 3). Therefore, there is an urgent need to identify individualized biomarkers and tailor targeted therapeutic strategies to optimize the outcome of ccRCC patients in clinical first-line therapy.

Cell death occurs in two main ways, accidental cell death is an uncontrollable biological process and programmed cell death (PCD) is a highly complex death program.PCD includes Apoptosis, Pyroptosis, Autophagy, Lysosome-dependent cell death, Necroptosis, Ferroptosis, Cuproptosis, Disulfidptosis, Entotic cell death, Parthanatos, Netotic cell death, Alkaliptosis and Oxeiptosis (4). A growing number of studies have shown that cell death is an important anticancer defense mechanism and therapeutic target (5). However, few studies have considered programmed cell death from the perspective of tumor immunotherapy. Although programmed cell death in the context of clear cell renal cell carcinoma has been extensively studied, the role of programmed cell death in this cancer type remains unclear (6). Therefore, there is a need to assess the prognostic importance of programmed cell death in clear cell renal cell carcinoma based on programmed cell death-associated genes and to predict which subtypes of clear cell renal cell carcinoma patients respond better to immunotherapy and chemotherapy.

Programmed cell death has been shown to exert an important influence during malignant tumor development and metastasis, and tumor cells need to overcome various forms of cell death in order to survive and develop (7). However, a comprehensive summary of the relationship between programmed cell death and ccRCC remains unknown. In this study, we established a new metric, the programmed cell death index (PCDI), by collecting genes during programmed cell death and thus by machine-learning methods to predict the prognosis of ccRCC patients and the effectiveness of therapeutic interventions. We performed *in vitro* experimental assays to evaluate the role of IDUA in ccRCC progression.

## Materials and methods

### Obtaining sequence data from patients with clear cell renal cell carcinoma

We collected key regulatory genes for 13 programmed cell death patterns as PCD-related genes from the GSEA gene set, KEGG, Hallmark, and review articles, and the final gene list is the tandem regulatory genes for 13 PCD patterns (4, 7–15). A final collection of 1217 PCD-related genes was included in the analysis (**Table S1**). We downloaded transcriptome profiles, corresponding clinical information and mutation data of ccRCC samples from The Cancer Genome Atlas (TCGA-KIRC) (16) species. In addition, we obtained DNA microcohort data and clinical characterization of ccRCC as a validation cohort from ICGC and GEO databases (ID: GSE36895 and GSE53757).

### Construction and validation of programmed cell death index (PCDI) and histological validation at the protein level

We used univariate Cox regression to assess the association of PCD-related genes with survival status in ccRCC patients, adjusting the cutoff P-value to 0.001. candidate genes were further narrowed down by Lasso and multivariate Cox regression methods to construct the most suitable signatures.The programmed cell death index (PCDI) was finally obtained for each patient by the following formula: programmed Cell death index (PCDI) = Coef(Gene 1) × Expr(Gene 1) + Coef(Gene 2) × Expr(Gene 2) +…… + Coef(Gene n) × Expr(Gene n). where Coef(Gene) represents the risk regression coefficient of Gene and Expr(Gene) represents the expression of Gene. Based on the median value of PCDI, we categorized ccRCC patients into high and low PCDI groups. We further searched the Human Protein Atlas database (https://www.proteinatlas.org/) (17) to obtain YBX3, BID, TCIRG1, IDUA, KDR, PYCARD, IFNG, and LRRK2 between clear cell renal cell carcinoma tissues and normal kidney tissues at the protein level for Histological validation.

### Unsupervised clustering of PCD-related genes

Based on PCD-related genes, we implemented consensus clustering using the R package “ConsensusClusterPlus” to identify ccRCC subtypes (18).

### Gene set enrichment analysis (GSEA)

We obtained reference genomes (Hallmark, c5go and c2kegg) from the Molecular Signatures Database (MSigDB). Hallmark, c5go and c2kegg analyses were performed using the R package “clusterProfiler”. Screening conditions were |NES| > 1, NOM p-value < 0.05.

### Somatic mutation analysis

To determine the mutational load of ccRCC, we used the R package “TCGAbiolinks” to obtain ccRCC mutation data (19). We then used the “maftools” package to analyze the mutation data and obtain the TMB for each patient. the results were visualized using a waterfall Figure (20). Based on the median, TMB was categorized into two groups, high TMB and low TMB, and the mutation characteristics were compared between different risk groups. In addition, KM curves were utilized to detect the relationship between TMB, different risk classes of TMB and prognosis of ccRCC.

### Immunomicroenvironment analysis

We used the R package “ESTIMATE” to perform tumor microenvironmental analysis to calculate tumor purity, ESTIMATE score, immune cell score and stroma score for each sample (21). The proportion of 22 types of immune infiltrating cells was calculated by the CIBERSORT algorithm (https://cibersort.stanford.edu/) (22). The single sample gene set enrichment analysis (ssGSEA) algorithm was used to quantify the relative proportions of immune cells and immune function.

### Drug response and immunotherapy response

We derived IC50 by ProPhetic algorithm to assess drug response to common chemotherapeutic treatments for ccRCC, comparing drug sensitivity to the above chemotherapeutic treatments in patients with high PCDI and low PCDI. We downloaded the gene expression data of cancer cells to different drugs at the Tumor Drug Sensitivity Multi-Organics (GDSC) database (https://www.cancerrxgene.org/) (23) and calculated IC50 values to assess patients’ responses to chemotherapeutic drugs. The Tumor Immune Dysfunction and Exclusion (TIDE) algorithm can be used to infer patient response to immunotherapy (24). In addition we downloaded anti-PD-1 and anti-CTLA4 IPS scoring data from ccRCC via the TCIA database (https://tcia.at/home) (25) to assess patient response to immune checkpoint inhibitors.The IMvigor210 study cohort evaluated the efficacy of atezolizumab (a PD-L1 targeting antibody) in 210 patients with locally advanced or metastatic uroepithelial cancer (26).The Liu study cohort evaluated 121 patients with metastatic melanoma treated with an anti-PD-1 inhibitor and the Kim study cohort of patients with metastatic gastric cancer treated with pembrolizumab (an anti-PD-1 inhibitor) (27). In addition, we collected genes that have been currently reported to be positively associated with immuno-efficacy and negatively associated with immuno-efficacy and analyzed them in association with PCDI (28–30).

### Bioinformatics analysis

Differential analysis of ccRCC and surrounding normal tissue was performed using the R package “limma” with a cutoff value set to log2 fold change (logFC) >1 and adjusted false discovery rate (FDR) <0.001 (31). Heatmap visualization was performed using the R package “pheatmap”. The R packages “rms” and “regplot” were used for the plotting of column line graphs and calibration curves. We calculated the tumor mutational load (TMB) of each patient and compared it between the high-risk and low-risk groups, and then applied the R package “Maftools” to explore the somatic mutation information in ccRCC patients. Principal component analysis (PCA) was performed using the “stats” package.

### Cell lines and cell culture

ACHN and 786−O cells were purchased from The Cell Bank of Type Culture Collection of The Chinese Academy of Sciences (Shanghai, China) and were cultured in accordance with the cell culture manual of the manufacturer. Passage time was less than 6 months and mycoplasma were eliminated according to the test report. ACHN cells were cultured in MEM medium (HyClone, Logan, UT, USA) and 786-O cells were cultured in RPMI-1640 medium (HyClone, Logan, UT, USA), the culture media contained 10% fetal bovine serum (FBS, HyClone) according to cell culture manual. All RCC cells were cultured in a humidified atmosphere of 5% CO_2_ at 37°C. When the cell fusion rate was 90%, the cells were digested with 1-ml trypsin for 5 minutes, then adding 1 ml of medium into the solution for neutralization. Collected by centrifugation, the cells were resuspended with culture media and passaged.

### Small interfering RNA (siRNA) transfections

ACHN and 786-O cell lines were cultured in 6-well plates for the transfection of the siRNA. When the cells confluence rate was 60%, the transfection complexes with IDUA siRNA (JTS scientific, Wuhan, China) were added into the culture solution by means of Lipofectamine™3000 (Invitrogen, USA) on the basis of the procedure in the instruction manual. The siRNA sequences were as follows (5′-3′): siIDUA-1# (sense ACUUUGAGGACAAGCAGCAGGUGUU and anti-sense AACACCUGCUGCUUGUCCUCAAAGU), siIDUA-2# (sense CAGCAGGUGUUUGAGUGGAAGGACU and anti-sense AGUCCUUCCACUCAAACACCUGCUG).

### Western blotting (WB)

Total cell lysates were extracted by adding the RIPA solution (RIPA: PMSF, 100:1; P0013B; Beyotime Institute of Biotechnology) containing protease and phosphatase inhibitors. The concentration of total cell protein was assessed with Enhanced BCA Protein Assay Kit (cat. no. P0010; Beyotime Institute of Biotechnology). Denatured protein (40 μg/lane) realized the protein separation on a 10% sodium dodecyl sulfate polyacrylamide gel electrophoresis (140 V, 50 minutes) gel, then target protein was transferred to the polyvinylidene fluoride membrane (350 mA, 90 minutes) by transfer. The membrane was soaked in a sealed container using 5% fat-free milk at 37°C for 1 hour to realize the protein blockade. The membrane was incubated overnight with the primary antibody in 5% fat-free milk at 4°C. The information of the primary antibodies in the WB were as follows: anti-IDUA (1:1000, 55158-1-AP, Proteintech) and anti-GAPDH (1:5000, 10494-1-AP, Proteintech). The membrane was washed three times (5 minutes each) using tris-buffered saline tween-20, then the membrane was incubated with the corresponding secondary antibody at 37°C for 1 hour. EasySee Western Blot kit (Beijing Transgen Biotech, Beijing, China) was used for detection following the experimental operation manual of manufacturer. Density measurements were carried out using ImageJ software (National Institute of Health, Bethesda, MD, USA), and the density of target protein bands were normalized with GAPDH.

### Cell viability assay

Cell viability was measured by colorimetric assay by means of Cell Counting Kit-8 (CCK-8) (Bimake, USA). 3 × 10^3^ cells were seeded in each well of 96-well plates. After 24 hours of transfection, CCK-8 working solution was added to the solution and incubated at 37°C for 1 hour. The absorbance of culture solution was measured at 450 nm using a plate reader (Model 680; Bio-Rad Laboratories) at 24, 48 and 72 h.

### 5−Ethynyl−2’−deoxyuridine (EdU) assay

ACHN and 786-O cells were transfected with IDUA siRNAs in 24-well plates for 48 h, with EdU (BeyoClick™, EDU-555, China) added to the medium (1:1000) to assess the cell proliferation. Cells were incubated for 2 hours at 37°C, after labeling, the culture medium was removed, and 1-ml fixative solution (4% paraformaldehyde) was added for fix cells at room temperature for 20 minutes. Cells were incubated at room temperature for 15 minutes with 1-ml permeate (0.3% Triton X-100) and the cells were treated with click reaction buffer at 37°C for 30 min, after which Hoechst solution was added for 15 min at room temperature. A fluorescence microscope (Olympus Corporation, Japan) was used to obtain the images to show the cell proliferation.

### Transwell assay

600 μL medium (10% FBS) was added to the bottom of the well, which is the lower chamber. 1 × 10^5^ cells were suspended in 200 μL serum-free medium and the suspension were seeded into the upper chamber (Corning, NY, USA). Then the chambers were placed into the 24-well plates. Cells were incubated at 37°C and 5% CO_2_ for 24 hours. After removing the remaining cells in the upper chamber using the swab, 4% paraformaldehyde was used to fix the cells for 10 minutes, and crystal violet stain was added for staining lasting for 10 minutes at room temperature. Cell migration was observed by means of optical microscopy, and ImageJ software lent itself to calculate the migration efficiency.

### Wound-healing assay

When the confluence rate of cells grew to 100% in the 6-well plates, a scratch was made in the center by means of the tip of the 1 mL pipette. Then cells were cultured with serum-free medium at 37˚C for 48 h. Cell images were gained with an microscope (EVOS XL system; AMEX12000; Thermo Fisher Scientific, Inc.). ImageJ software were applied to calculate the wound area.

### Statistical analysis

Survival curves were plotted using the Kaplan-Meier method to compare the difference in survival between the two groups. p-value ≤ 0.05 was considered statistically significant. All statistical analyses were performed by R.

## Results

### Clustering of programmed cell death-related genes

**Figure 1:**
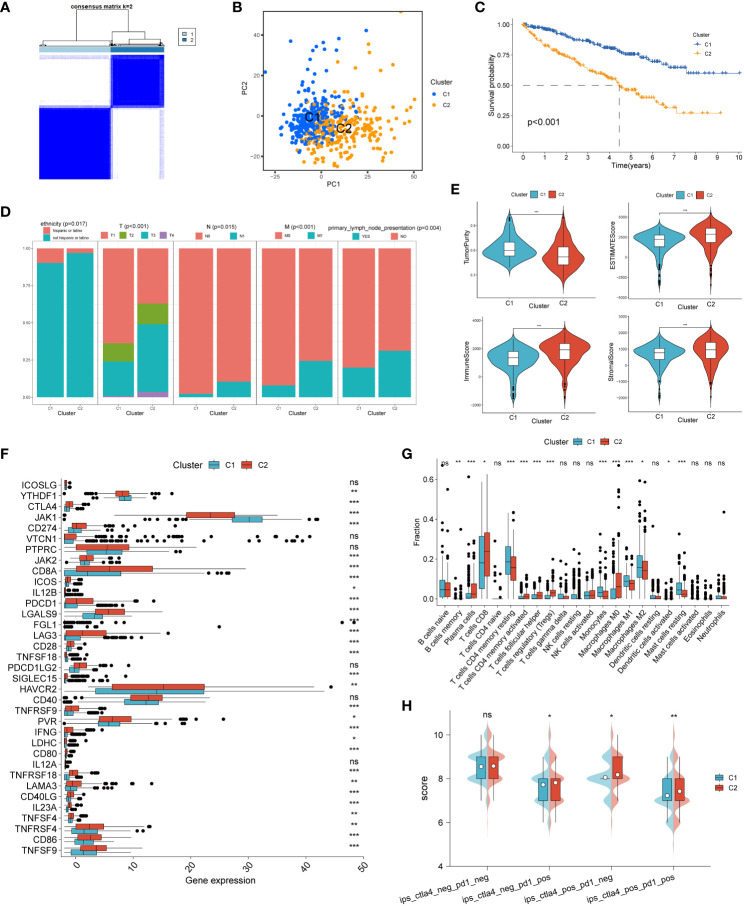
Unsupervised cluster analysis of programmed cell death genes. **(A)** When k=2, ccRCC patients were divided into two clusters based on programmed cell death-related genes. **(B)** Principal component analysis (PCA) plot based on PCD-related genes. **(C)** Kaplan-Meier curves showing the prognosis of ccRCC patients in two clusters. **(D)** Proportion of clinicopathologic features between two clusters. **(E)** Differences in tumor purity, ESTIMATEScore, immune score and stromal score between the two clusters. Differences between common immune checkpoints **(F)** and immune cells **(G)** between the two clusters. **(H)** between the two clusters to PD1- or no-CTLA4 blockers, PD1 blockers, CTLA4 blockers, and PD1-CTLA4 co-blockers. *p < 0.05 ,**p < 0.01, ***p < 0.001. ns, non significance.

We performed consensus clustering analysis to explore ccRCC subtypes by collecting PCD-related genes (1217 genes in total). We found that differences between subgroups were most significant when k=2, indicating that ccRCC patients could be well categorized into two clusters (**Figure 1A**). The results of principal component analysis (PCA) showed that patients with renal clear cell carcinoma could be categorized into two clusters based on PCD-related genes (**Figure 1B**). We found a significant difference in patient overall survival (OS) between the two clusters (p < 0.001), with C1 associated with patients with a good prognosis and C2 associated with patients with a poor prognosis (**Figure 1C**). By comparing the differences in clinicopathologic characteristics between the two clusters, including ETHNICITY, T, N, M, and whether lymph node metastasis occurred. We found that not Hispanic or latino, T, N, M staging and occurrence of lymph node metastasis were higher in C2 than in C1, suggesting a higher malignant phenotype in C2 patients (**Figure 1D**).

We then analyzed the immune profile between the two clusters. We found that the tumor purity of C2 was significantly lower than that of C1, while the ESTIMATEScore, immune score and stromal score of C2 were significantly higher than that of C1, suggesting a high level of immune infiltration in the C2 phenotype compared to C1 (**Figure 1E**). Analysis of the differences in the expression of common immune checkpoints between the two clusters showed that most of the immune checkpoints were expressed significantly higher in type C2 than in type C1 (**Figure 1F**). The infiltration abundance of immune cells was assessed by the CIBERSORT algorithm, and the results showed that the level of immune cell infiltration was higher in type C2 (**Figure 1G**). Finally, the effect of the two clusters on the response to immunotherapy was assessed by the IPS score, and we found that C2 was significantly more effective than C1 against anti-PD-1 and/or anti-CTLA-4 inhibitors (**Figure 1H**).

### Construction of programmed cell death index (PCDI) in ccRCC patients

**Figure 2:**
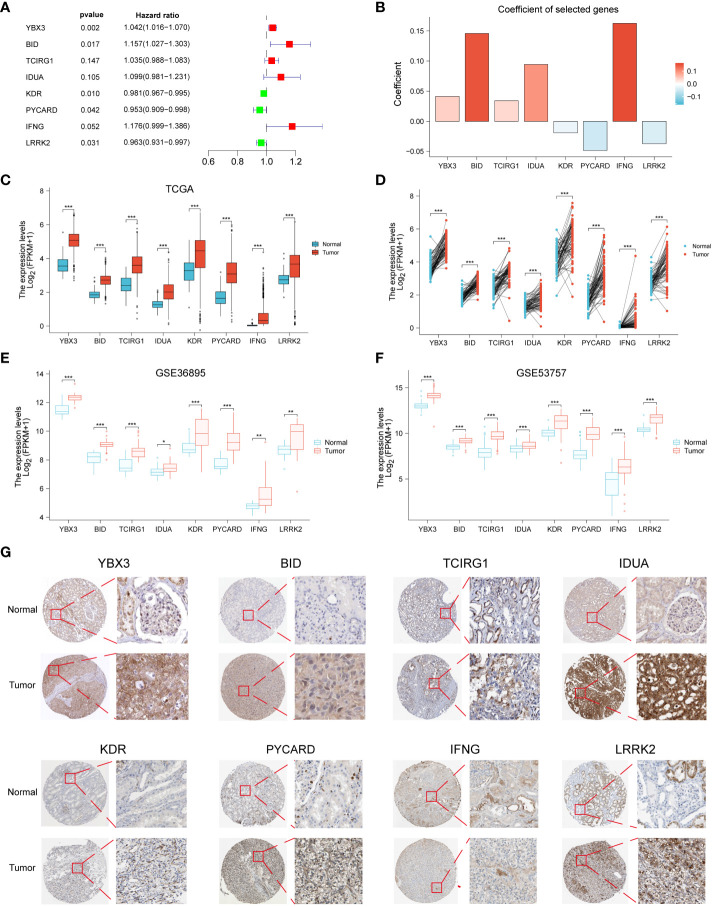
Construction of PCDI in ccRCC patients and validation of immunohistochemical staining results. Forest plots **(A)** and coefficient histograms **(B)** of the eight genes for which PCDI was constructed. YBX3, BID, TCIRG1, IDUA, KDR, PYCARD, IFNG, and LRRK2 were found to be different in the TCGA dataset in unpaired samples **(C)** and paired samples **(D)** of tumor tissues and normal tissues as well as in the GSE36895 **(E)** and GSE53757 **(F)** Differences between. **(G)** Immunohistochemical staining results of YBX3, BID, TCIRG1, IDUA, KDR, PYCARD, IFNG and LRRK2 in normal and cccRCC tissues. *p < 0.05 ,**p < 0.01, ***p < 0.001. ns, non significance.

We identified 8 genes and thus constructs with programmed cell death and its derived PCD by one-way Cox regression, lasso and multifactorial Cox regression analyses (**Figure 2A**). Our model derived the programmed cell death index (PCDI) for each patient by the following equation. PCDI= (0.0413*YBX3 exp.) + (0.1458*BID exp.) + (0.0341*TCIRG1 exp.) + (0.0943*IDUA exp.) + (-0.0192*KDR exp.) + (-0.0485*PYCARD exp.) + (0.1625*IFNG exp.) + (-0.0373*LRRK2 exp) (**Figure 2B**). We then explored the expression of PCDI genes in unpaired and paired samples of tumor and normal tissues in the TCGA-KIRC cohort, and showed that all eight genes comprising PCDI were significantly upregulated and expressed in tumor tissues (p < 0.05) (**Figures 2C, D**). We validated these results by two GEO datasets (GSE36895 and GSE53757), and the results were consistent (**Figure 2E, F**). The expression of the eight genes comprising PCDI at the protein level was analyzed by immunohistochemical staining of the HPA database, and we found that compared with normal tissues, the eight genes comprising PCDI stained more deeply immunohistochemically in tumor tissues, suggesting that the content of these eight proteins was higher than that in normal tissues in ccRCC (**Figure 2G**).

### External dataset validation and clinical relevance of PCDI

**Figure 3:**
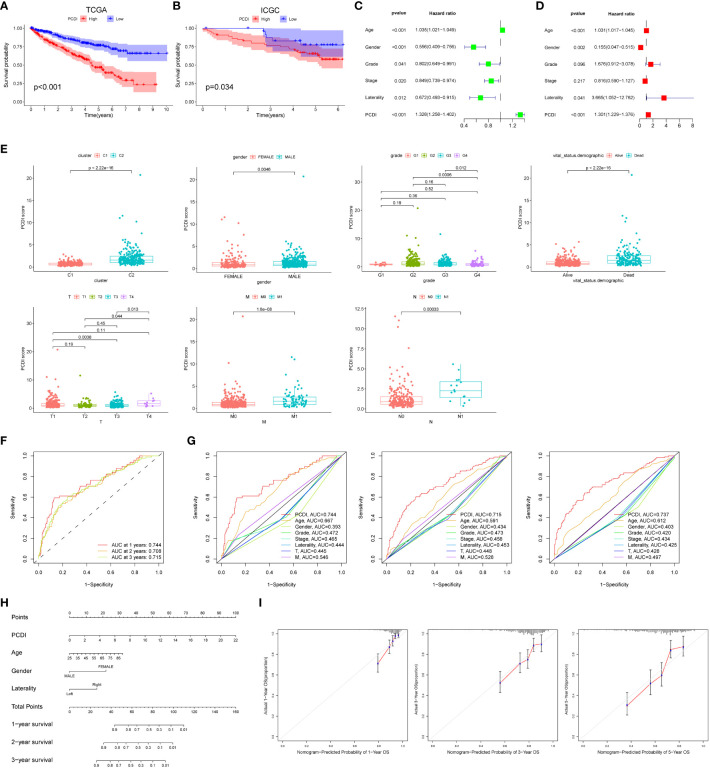
External dataset validation and clinical correlations of PCDI. Kaplan-Meier survival curves comparing high/low PCDI for the TCGA dataset **(A)** and the ICGC external dataset **(B)**. Univariate **(C)** and multivariate **(D)** Cox regression analysis of PCDI and other clinical traits. **(E)** Differences between PCDI between common clinical traits. **(F)** ROC curves of PCDI at 1, 3 and 5 years. **(G)** ROC curves of PCDI compared with other clinical traits at 1, 3 and 5 years. **(H)** Column line graphs predicting prognosis in ccRCC patients. **(I)** Calibration curves for the probability of overall survival at 1, 3 and 5 years in the TCGA cohort.

We found that patients in the high PCDI group had a poorer prognosis and were more likely to have a higher mortality rate through Kaplan-Meier survival curves (p < 0.05, **Figure 3A**). Subsequently, we used the ICGC dataset as a validation cohort, and Kaplan-Meier analysis showed that patients in the high PCDI group were more likely to have poorer overall survival (**Figure 3B**). The results of univariate and multivariate Cox regression analyses performed showed that the p-value of PCDI was less than 0.05, suggesting that PCDI can be an independent prognostic factor in patients with ccRCC (**Figures 3C, D**). To further validate the clinical significance of PCDI, we analyzed the differences in PCDI between different clinical characteristics and showed that PCDI was higher in patients with C2, male, high grading, high T stage, M1 and N1 stage, suggesting that the higher our PCDI, the closer ccRCC was to advanced stage (**Figure 3E**). Analysis of survival curves stratified by clinical characteristics showed that PCDI significantly differentiated the prognosis of each clinical subgroup, with patients in the high PCDI group having a worse prognosis (**Figure S1**). We evaluated the area under the curve (AUC) values of the TCGA cohort and compared them with other clinical traits, and the results showed that PCDI had high accuracy in predicting 1-, 3-, and 5-year survival in patients with ccRCC and was a better predictor of patient survival than other clinical traits (**Figures 3F, G**). With the results of univariate and multivariate Cox regression analyses, we modeled column-line plots for ccRCC patients to assess patient prognosis, with age, gender, Laterality, and PCDI included in the models (**Figure 3H**). The calibration curves of the column-line plots showed that the predicted 1-, 3-, and 5-year survival rates were more consistent with the actual survival rates at the reference line, suggesting that the constructed column-line plots could predict patient prognosis well (**Figure 3I**).

### PCDI predicts prognosis extended from ccRCC to pan-cancer

**Figure 4:**
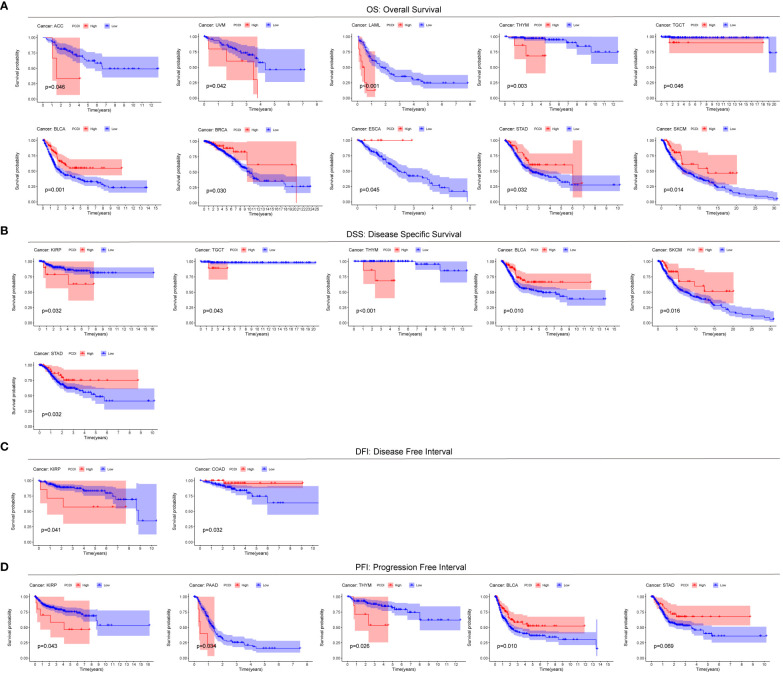
Predictive value of PCDI in other cancers. Comparison of Overall Survival (OS) **(A)**, Disease Specific Survival (DSS) **(B)**, Disease Free Interval (DFI) **(C)**, and Progression Free Interval (PFI) **(D)**.

To explore the generalization of PCDI to other cancers, we used the modeling equations for PCDI described above to calculate PCDI values for patients with the other 32 cancer types in TCGA and to plot Kaplan-Meier survival curves for the high/low PCDI group. For Overall Survival (OS), patients in the high PCDI group in ACC, UVM, LAML, THYM, and TGCT had a poorer prognosis, whereas patients in the low PCDI group in BLCA, BRCA, ESCA, STAD, and SKCM had a poorer prognosis (**Figure 4A**). For Disease Specific Survival (DSS), patients in the high PCDI group in KIRP, TGCT, and THYM had poor Disease Specific Survival, while patients in the low PCDI group in BLCA, SKCM, and STAD had poor Disease Specific Survival (**Figure 4B**). For Disease Free Interval (DFI), patients in the high PCDI group in KIRP had poor Disease Free Interval, whereas patients in the low PCDI group in COAD had poor Disease Free Interval (**Figure 4C**). For Progression Free Interval (PFI), patients in the high PCDI group in KIRP, PAAD, and THYM had poor Progression Free Interval, whereas patients in the low PCDI group in BLCA and STAD had poor Progression Free Interval (**Figure 4D**). The above results suggest that PCDI not only has a good effect in predicting the prognosis of ccRCC (KIRC), but also has a predictive prognostic value in other cancers.

### Tumor microenvironment dissection based on programmed cell death index

**Figure 5:**
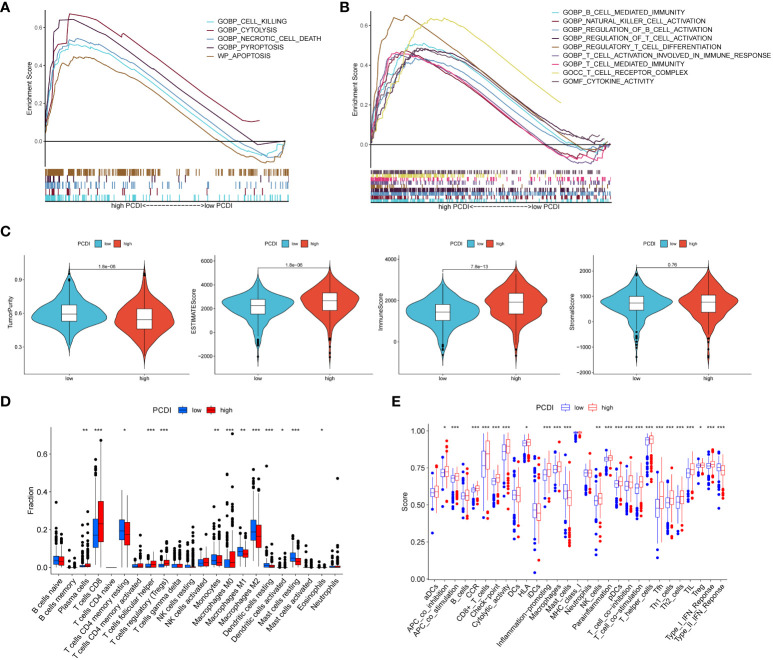
Assessment of tumor microenvironment based on programmed cell death index. **(A, B)** GSEA analysis of patients in the high PCDI group. **(C)** Differences in tumor purity, ESTIMATEScore, immune score and stromal score between high/low PCDI groups. **(D)** The ssGSEA algorithm assesses differences in immune cells and immune function between patients in the high/low PCDI group. **(E)** CIBERSORT algorithm to assess immune cell differences between patients in the high/low PCDI group. *p < 0.05 ,**p < 0.01, ***p < 0.001. ns, non significance.

In order to investigate the regulatory pathways of tumorigenesis in the high PCDI group, we performed GSEA analysis of tumors in the high PCDI group, which showed that the high PCDI group was significantly enriched in CELL Killing, CYTOLYSIS, Necrotic cell death, Pyroptosis, and Apoptosis (p < 0.05) (**Figure 5A**). In addition, tumors in the high PCDI group were significantly enriched (p < 0.05) in immune function pathways such as B cell mediated immunity, NK cell activation, T cell activation and T cell mediated immunity, suggesting that our high PCDI group and tumor immune microenvironment closely associated with the tumor immune microenvironment (**Figure 5B**).

Using the ESTIMATE algorithm, we found that the high PCDI group had lower tumor purity but higher immune scores and stroma scores (**Figure 5C**). ssGSEA algorithm results showed that patients in the high PCDI group had better immune cell infiltration and immune-related functions compared to the low PCDI group (**Figure 5D**). cIBERSORT algorithm showed that the high PCDI group The level of immunostimulatory CD8 T cells was significantly higher than that of the low PCDI group, while the level of immunosuppressive M2-type macrophages was significantly lower in the high PCDI group, which suggested that our high PCDI group had high immune infiltration characteristics (**Figure 5E**).

### Efficacy of PCDI in predicting immunotherapy outcome

**Figure 6:**
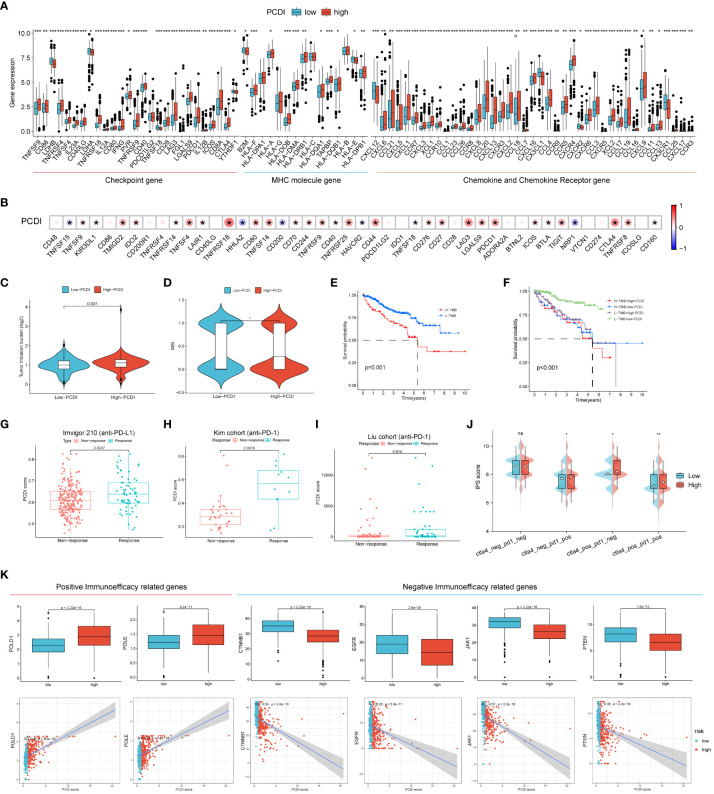
Efficacy of programmed cell death index in predicting immunotherapy outcome. **(A)** Differences in the expression levels of common immune checkpoints, MHC molecules, cytokines and cytokine receptors between high/low PCDI groups. **(B)** Correlation between PCDI and common immune checkpoints. Differences between TMB **(C)** and MSI **(D)** between high and low PCDI groups. **(E, F)** TMB and survival curves associated with PCDI. External immunotherapy datasets Imvigor210 **(G)**, Kim cohort **(H)** and Liu cohort **(I)** validate PCDI for immunotherapy effect prediction. **(J)** between high-PCDI and low-PCDI groups to PD1- or no-CTLA4 blockers, PD1 blockers, CTLA4 blockers, and PD1-CTLA4 co-blockers. **(K)** Association between positive immunotherapy efficacy-related genes and negative immunotherapy efficacy-related genes with PCDI. *p < 0.05 ,**p < 0.01, ***p < 0.001. ns, non significance.

To further explore the relationship between PCDI and the immune microenvironment, we compared the differences in the expression levels of common immune checkpoints, MHC molecules, cytokines, and cytokine receptors between the high/low PCDI groups, and the results showed that the expression levels of immune checkpoints, MHC molecules, cytokines, and cytokine receptors of patients in the high PCDI group were significantly higher than those in the low PCDI group (p < 0.05, **Figure 6A**). Then we analyzed the correlation between PCDI and common immune checkpoints, and the results showed that most of the immune checkpoints were significantly positively correlated with PCDI (p < 0.05, **Figure 6B**). These results suggest that the tumors in the high PCDI group showed “hot” tumor characteristics, and the efficacy of immune checkpoint inhibitor therapy in the high PCDI group may be better than that in the low PCDI group. By analyzing the association between TMB, MSI and PCDI, we found that patients in the high PCDI group showed higher TMB and MSI (**Figures 6C, D**). In addition, the prognosis of patients in the high TMB group was worse than that of patients with low TMB. ccRCC patients were divided into four groups after the combination of TMB and PCDI, and we found that the prognosis of patients in the high TMB+high PCDI group, the high TMB+low PCDI group, and the low TMB+high PCDI group were all similarly worse than that of patients in the high TMB+high PCDI group (**Figures 6E, F**). We also examined the mutation rates in ccRCC in the cBioPortal database for the eight genes from which PCDI was constructed and found a higher mutation rate of 15% for LRRK2 and a lower mutation rate of 4% for PYCARD compared to the other six genes from which PCDI was constructed (**Figure S2**).

Subsequently, we collected external immunotherapy datasets to validate PCDI to predict immunotherapy efficacy, and the results showed that PCDI was significantly higher in patients in the immunotherapy-responsive group than in the non-responsive group in Imvigor210 (anti-PD-L1), Kim cohort (anti-PD-1), and Liu cohort (anti-PD-1) patients (**Figures 6G–I**). We then explored the association between PCDI group and immunophenotypic core (IPS) by assessing immunogenicity to predict patient response to immune checkpoint blockade (anti-PD1 and/or anti-CTLA4), and we found that IPS scores were higher in the high PCDI group, suggesting that patients in the high PCDI group may be better responders to immunotherapy (**Figure 6J**). We also compared the association between positive and negative immune efficacy-related genes and PCDI, and showed that PCDI was significantly positively associated with positive immune efficacy-related genes (POLD1 and POLE) and significantly negatively associated with negative immune efficacy-related genes (CTNNB1, EGFR, JAK1 and PTEN) (**Figure 6K**). All these results indicated that PCDI could better predict the immunotherapy effect, and the high PCDI group had better effect on immunotherapy.

### Efficacy of PCDI in predicting drug sensitivity

**Figure 7:**
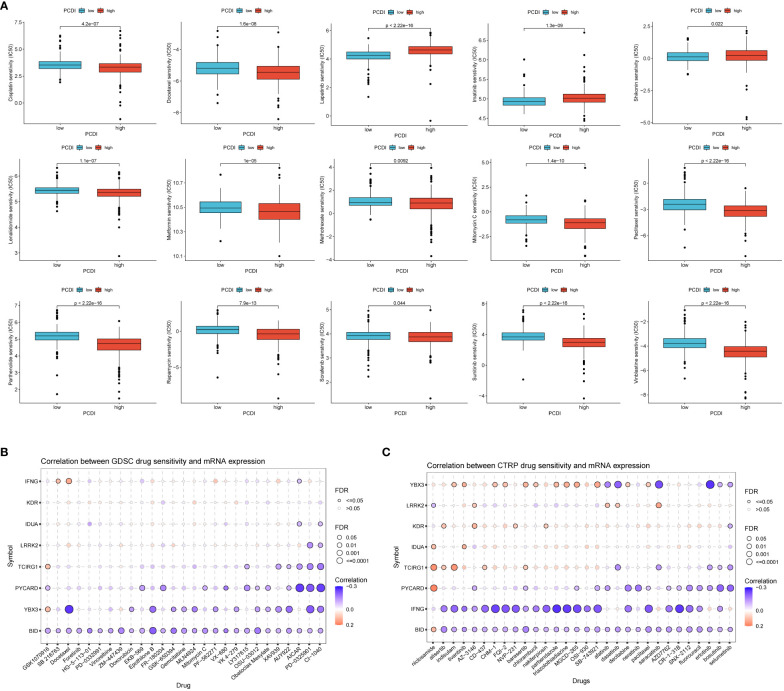
Value of programmed cell death index in predicting drug sensitivity. **(A)** Differences in response to common chemotherapeutic drugs between the high and low PCDI groups. the GDSC database **(B)** and the CTRP database **(C)** analyzed the relationship between drug sensitivity and mRNA expression of the eight genes that construct the PCDI.

To explore the association between PCDI and drug sensitivity, we calculated half-maximal inhibitory concentration (IC50) values for common drugs in ccRCC samples and compared them with those between PCDI subgroups. We found that many common chemotherapeutic agents for renal cell carcinoma were significantly different between the high/low PCDI groups (p < 0.05), and the IC50 values for Docetaxel were significantly lower in the high PCDI group than in the low PCDI group, suggesting that patients in the high PCDI group may have a better response to Docetaxel-based chemotherapy.The IC50 values for Lapatinib were significantly lower in the low PCDI group was significantly lower than that in the high PCDI group, suggesting that patients in the low PCDI group had a better response to Lapatinib-based chemotherapy (**Figure 7A**). Then, we analyzed the relationship between drug sensitivity and mRNA expression of the eight genes constructing PCDI using the GDSC and CTRP databases; positive correlation indicated that gene expression was associated with drug resistance, while negative correlation indicated that gene expression was associated with drug sensitivity. The results indicated that BID and YBX3 gene expression was associated with most drug sensitivities (**Figures 7B, C**).

### Knock-down of IDUA inhibited RCC cell proliferation and migration

**Figure 8:**
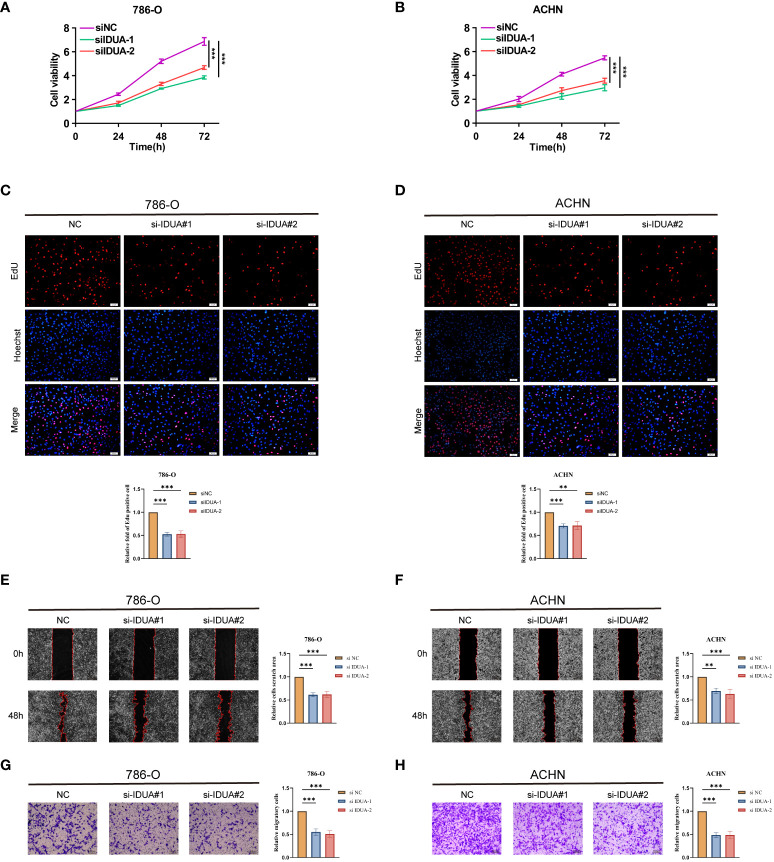
IDUA promotes proliferation and migration of RCC cells. **(A, B)** Cell proliferation of 786-O or ACHN cells transfected with control or si-IDUA was measured by CCK8. **(C, D)** Edu assay to show the cell proliferation of knock-down IDUA cells compared with the corresponding control cells. **(E, F)** Transwell assay to show the cell metastasis of knock-down IDUA cells compared with the corresponding control cells. **(G, H)** Wound-healing assay to show the cell metastasis of knock-down IDUA cells compared with the corresponding control cells. *p < 0.05 ,**p < 0.01, ***p < 0.001.

To confirm the biological function of IDUA in RCC, we knocked down IDUA using two siRNAs in ACHN and 786-O cells. Western blotting showed that IDUA could be effectively silenced by two independent siRNAs (**Figure S3**). The proliferation of ACHN and 786-O cells was noticeably decreased after down-regulation of IDUA expression compared with the corresponding control cells in the CCK8 assay (**Figures 8A, B**). In addition, EDU incorporation analysis also showed that the proportion of EDU-positive ACHN and 786-O cells was significantly reduced in the IDUA-interfered group compared with the corresponding control cells (**Figures 8C, D**). The results of transwell assay showed that interference with IDUA crippled the migratory capacity of ACHN and 786-O cells (**Figures 8E, F**). Subsequently, in the wound-healing assay, we found that IDUA knocking-down decelerated wound closure speed in both ACHN and 786-O cells (**Figures 8G, H**). These results indicated that IDUA could promote renal cell carcinoma cell proliferation and migration *in vitro*. IDUA is expected to be a potential target for the treatment of renal cell carcinoma.

## Discussion

An increasing number of studies have shown that cell death is an important anti-cancer defense mechanism and therapeutic target. In this study, we established a programmed cell death index (PCDI) constructed from eight programmed cell death-related genes (TBX3, BID, TCIRG1, IDUA, KDR, PYCARD, IFNG, and LRRK2) based on the data of 13 different programmed cell death modalities. The PCDI can be used as a marker to classify the subtypes of clear cell renal cell carcinoma and can be effectively used to predict the prognosis and immunotherapy outcome of patients with clear cell renal cancer. effectively used to predict the prognosis and immunotherapy outcome of patients with clear cell renal cell carcinoma. Finally, we validated the effect of IDUA on clear cell renal cell carcinoma *in vitro*.

To determine the association between PCDI and clear cell renal cell carcinoma, we first found that the high PCDI group was strongly associated with immune cells (e.g., CD8+ T cells, NK cells, and B cells) by gene set enrichment analysis. Then we assessed by ESTIMATE algorithm, ssGSEA algorithm, and CIBERSORT algorithm that patients in the high PCDI group were indeed associated with high levels of immune infiltration. We also found that most of the common immune checkpoints, MHA molecules, cytokines and receptors were significantly up-regulated in the high PCDI group by comparing the expression levels of these molecules between the high PCDI group and the low PCDI group. In addition, TMB and MSI have an important role in predicting patient immunotherapy (32). Current studies have shown that high immune cell infiltration and high immune checkpoint expression are characteristic of “hot” tumors, which are effective for immunotherapy (33, 34). TMB and MSI were also high in the high PCDI group, suggesting that our patients in the high PCDI group showed “hot” tumor characteristics that may be effective for immunotherapy. We then verified these results with external immunotherapy datasets Imvigor210 (anti-PD-L1), Kim cohort (anti-PD-1), and Liu cohort (anti-PD-1), and found that the PCDI values of the patients in the immunotherapy-responsive group were significantly higher than those of the patients in the non-responsive group. On the other hand, by evaluating the IC50 values of common chemotherapeutic drugs differing between high/low PCDI groups and calculating the association between the expression levels of genes involved in the construction of PCDI and drug sensitivities, we found that the high/low PCDI groups could significantly differentiate the sensitivities to common chemotherapeutic drugs in ccRCC patients. The above results indicate that PCDI can effectively assess the sensitivity to chemotherapeutic drugs and immunotherapy efficacy in patients with clear cell renal cell carcinoma, which is important for the future precision treatment of patients with clear cell renal cell carcinoma.

The PCDI index was constructed by the inclusion of eight genes, including TBX3, BID, TCIRG1, IDUA, KDR, PYCARD, IFNG, and LRRK2, all of which are highly expressed in clear cell renal cell carcinomas. T-box Transcription Factor 3 (TBX3), a transcription factor that not only acts as a regulator in many critical organs, such as the heart, breast, and limbs. It is also closely related to many cancers and stem cell maintenance (35).BH3 Interacting Domain Death Agonist (BID) is a pro-apoptotic factor of the Bcl-2 family that encodes a death agonist and can regulate apoptosis by forming a heterodimer with the agonist BAX or the antagonist BCL2 (36). TCIRG1, also known as the V-ATPase-a3 subunit, is essential in a variety of biological processes such as cellular metabolism, membrane trafficking, and intracellular signaling through its dependent acidification (37). KDR encodes the formative protein VEGFR2, which, as an important tyrosine transmembrane protein, plays an important role in regulating endothelial cell proliferation and migration, regulating angiogenesis, and other biological processes. play an important role in the regulation of endothelial cell proliferation and migration, and the regulation of angiogenesis. In addition, VEGFR2 has been shown to be aberrantly expressed in many malignant tumors and is associated with tumor progression and drug resistance, therefore, inhibitors targeting VEGFR2 are now considered to be promising and effective cancer therapeutic agents (38).Apoptosis-associated speck-like protein containing a caspase recruit domain (ASC) protein, encoded by the PYCARD gene, is involved in inflammatory and immune-related biological processes by aggregating ASC speckles during inflammatory vesicle activation due to pyroptosis (39).IFNG is a cytokine that regulates immune processes, and also possesses antimicrobial and anticancer activities. IFNG is a cytokine that regulates immune processes and has antimicrobial and anticancer activities, e.g., it induces iron death in tumor cells and inhibits tumor growth (40, 41). Leucine Rich Repeat Kinase 2 (LRRK2), a kinase with a multistructural domain and GTP activity, regulates autophagy, and has been of great interest in a wide range of diseases (42).

Although our constructed programmed cell death index (PCDI) closely reflects the prognosis of clear cell renal carcinoma as well as predicts chemo-sensitivity and immunotherapy efficacy, and predicts the prognosis and immunotherapy efficacy of a wide range of cancers other than clear cell renal cell carcinoma. However, certain limitations still exist in this study. First, the data for our analysis were obtained from public databases, which may lead to some case selection bias in case selection. It is also still necessary to collect a large amount of clinical case data for evaluation to further validate the accuracy of our findings. Finally, further *in vivo* and *in vitro* experiments are needed to validate the specific molecular mechanisms of the genes constructing the PCDI index in clear cell renal cell carcinoma progression.

## Conclusion

In summary, based on the comprehensive analysis of 13 programmed cell death-related genes in clear cell renal cell carcinoma, we conclude that PCDI can reliably and effectively predict the prognosis and immunotherapeutic efficacy of clear cell renal cell carcinoma. The present study identifies new prognostic and therapeutic biomarkers and targeted small molecule drugs for ccRCC from the perspective of many different programmed cell deaths, which provides useful clues for future precision treatment of clear cell renal cell carcinoma.

## Data availability statement

The original contributions presented in the study are included in the article/**Supplementary Material**, further inquiries can be directed to the corresponding author/s.

## Ethics statement

Ethical approval was not required for the studies on humans in accordance with the local legislation and institutional requirements because only commercially available established cell lines were used.

## Author contributions

ZQW: formal analysis, data curation, conceptualization, writing—original draft. MYJ: formal analysis, visualization, writing—original draft. PX: software, supervision, investigation, writing—review and editing. HZ: writing—review and editing, supervision, project administration, funding acquisition. All authors contributed to the article and approved the submitted version.

## Supplementary material

Supplementary Figure 1 Prognosis curve among clinical subgroups.

Supplementary Figure 2 Association of PCDI with somatic mutations. **(A)** Mutation rates of the eight genes for which PCDI was constructed. Waterfall plots of somatic mutations in the high PCDI group **(B)** and low PCDI group **(C)**.

Supplementary Figure 3 Western blotting for validating the knock-down effect of IDUA gene in RCC cell lines 786-O and ACHN.

## Glossary

**Table:**

TCGA	The Cancer Genome Atlas
GEO	Gene Expression Omnibus
BCa	Bladder cancer
GTEx	Genotype-Tissue Expression Program
WGCNA	Weighted Gene co-expression Network Analysis
PMGI	PD-L1 multidimensional regulatory index
HR	Hazard Ratio
ROC	Receiver Operating Characteristic
PD-1	Programmed cell death 1
PD-L1	Programmed cell death 1 ligand 1
CTLA4	Cytotoxic T-lymphocyte-associated protein 4
ICB	Immune checkpoint blockade
OS	Overall survival
ROC	Receiver Operation Characteristic
NES	Normalized enrichment score
TMB	Tumor mutation burden
IC50	half maximal inhibitory concentration
GDSC	Genomics of Drug Sensitivity in Cancer
PPI	protein–protein interaction
KM	Kaplan–Meier
ssGSEA	single-sample gene set enrichment analysis
TIDE	tumor immune dysfunction and exclusion
GSEA	gene set enrichment analysis
GO	Gene Ontology
KEGG	Kyoto Encyclopedia of Genes and Genomes
LAG3	Lymphocyte Activating 3
PFS	Progression-free survival
DSS	Disease-free survival
TP53	Tumor Protein P53
ACC	Adrenocortical carcinoma
BLCA	Bladder Urothelial Carcinoma, BRCA, Breast invasive carcinoma
CESC	Cervical squamous cell carcinoma and endocervical adenocarcinoma
CHOL	Cholangiocarcinoma
COAD	Colon adenocarcinoma
DLBC	Lymphoid Neoplasm Diffuse Large B-cell Lymphoma
ESCA	Esophageal carcinoma
GBM	Glioblastoma multiforme
HNSC	Head and Neck squamous cell carcinoma
KICH	Kidney Chromophobe
KIRC	Kidney renal clear cell carcinoma
KIRP	Kidney renal papillary cell carcinoma
LAML	Acute Myeloid Leukemia
LGG	Brain Lower Grade Glioma
LIHC	Liver hepatocellular carcinoma
LUAD	Lung adenocarcinoma
LUSC	Lung squamous cell carcinoma
MESO	Mesothelioma
OV	Ovarian serous cystadenocarcinoma
PAAD	Pancreatic adenocarcinoma
PCPG	Pheochromocytoma and Paraganglioma
PRAD	Prostate adenocarcinoma
READ	Rectum adenocarcinoma
SARC	Sarcoma
SKCM	Skin Cutaneous Melanoma
STAD	Stomach adenocarcinoma
TGCT	Testicular Germ Cell Tumors
THCA	Thyroid carcinoma
THYM	Thymoma
UCEC	Uterine Corpus Endometrial Carcinoma
UCS	Uterine Carcinosarcoma
UVM	Uveal Melanoma
CNV	Copy number variation

## References

[1] Siegel RL Miller KD Jemal A. Cancer statistics, 2019. CA: Cancer J Clin (2019) 69(1):7–34. doi: 10.3322/caac.21551 30620402

[2] Choueiri TK Halabi S Sanford BL Hahn O Michaelson MD Walsh MK. Cabozantinib versus sunitinib as initial targeted therapy for patients with metastatic renal cell carcinoma of poor or intermediate risk: the alliance A031203 CABOSUN trial. J Clin Oncol (2017) 35(6):591–7. doi: 10.1200/JCO.2016.70.7398 PMC5455807 28199818

[3] Motzer RJ Barrios CH Kim TM Falcon S Cosgriff T Harker WG. Phase II randomized trial comparing sequential first-line everolimus and second-line sunitinib versus first-line sunitinib and second-line everolimus in patients with metastatic renal cell carcinoma. J Clin Oncol (2014) 32(25):2765–72. doi: 10.1200/JCO.2013.54.6911 PMC5569681 25049330

[4] Tang D Kang R Berghe TV Vandenabeele P Kroemer G. The molecular machinery of regulated cell death. Cell Res (2019) 29(5):347–64. doi: 10.1038/s41422-019-0164-5 PMC6796845 30948788

[5] Carneiro BA El-Deiry WS. Targeting apoptosis in cancer therapy. Nat Rev Clin Oncol (2020) 17(7):395–417. doi: 10.1038/s41571-020-0341-y 32203277 PMC8211386

[6] Atkins MB Tannir NM. Current and emerging therapies for first-line treatment of metastatic clear cell renal cell carcinoma. Cancer Treat Rev (2018) 70:127–37. doi: 10.1016/j.ctrv.2018.07.009 30173085

[7] Su Z Yang Z Xu Y Chen Y Yu Q. Apoptosis, autophagy, necroptosis, and cancer metastasis. Mol cancer. (2015) 14:48. doi: 10.1186/s12943-015-0321-5 25743109 PMC4343053

[8] Tang R Xu J Zhang B Liu J Liang C Hua J. Ferroptosis, necroptosis, and pyroptosis in anticancer immunity. J Hematol Oncol (2020) 13(1):110. doi: 10.1186/s13045-020-00946-7 32778143 PMC7418434

[9] Stockwell BR Friedmann Angeli JP Bayir H Bush AI Conrad M Dixon SJ. Ferroptosis: A regulated cell death nexus linking metabolism, redox biology, and disease. Cell (2017) 171(2):273–85. doi: 10.1016/j.cell.2017.09.021 PMC5685180 28985560

[10] Tsvetkov P Coy S Petrova B Dreishpoon M Verma A Abdusamad M. Copper induces cell death by targeting lipoylated TCA cycle proteins. Sci (New York NY). (2022) 375(6586):1254–61. doi: 10.1126/science.abf0529 PMC9273333 35298263

[11] Wen S Niu Y Lee SO Chang C. Androgen receptor (AR) positive vs negative roles in prostate cancer cell deaths including apoptosis, anoikis, entosis, necrosis and autophagic cell death. Cancer Treat Rev (2014) 40(1):31–40. doi: 10.1016/j.ctrv.2013.07.008 23993415 PMC3833078

[12] Fatokun AA Dawson VL Dawson TM. Parthanatos: mitochondrial-linked mechanisms and therapeutic opportunities. Br J Pharmacol (2014) 171(8):2000–16. doi: 10.1111/bph.12416 PMC3976618 24684389

[13] Song X Zhu S Xie Y Liu J Sun L Zeng D. JTC801 induces pH-dependent death specifically in cancer cells and slows growth of tumors in mice. Gastroenterology (2018) 154(5):1480–93. doi: 10.1053/j.gastro.2017.12.004 PMC5880694 29248440

[14] Scaturro P Pichlmair A. Oxeiptosis: a discreet way to respond to radicals. Curr Opin Immunol (2019) 56:37–43. doi: 10.1016/j.coi.2018.10.006 30342374

[15] Zhao R Kaakati R Lee AK Liu X Li F Li CY. Novel roles of apoptotic caspases in tumor repopulation, epigenetic reprogramming, carcinogenesis, and beyond. Cancer metastasis Rev (2018) 37(2-3):227–36. doi: 10.1007/s10555-018-9736-y PMC6204284 29858742

[16] Liu J Lichtenberg T Hoadley KA Poisson LM Lazar AJ Cherniack AD. An integrated TCGA pan-cancer clinical data resource to drive high-quality survival outcome analytics. Cell (2018) 173(2):400–16.e11. 29625055 10.1016/j.cell.2018.02.052 PMC6066282

[17] Uhlén M Fagerberg L Hallström BM Lindskog C Oksvold P Mardinoglu A. Proteomics. Tissue-based map of the human proteome. Sci (New York NY). (2015) 347(6220):1260419. 10.1126/science.1260419 25613900

[18] Wilkerson MD Hayes DN. ConsensusClusterPlus: a class discovery tool with confidence assessments and item tracking. Bioinf (Oxford England). (2010) 26(12):1572–3. 10.1093/bioinformatics/btq170 PMC2881355 20427518

[19] Colaprico A Silva TC Olsen C Garofano L Cava C Garolini D. TCGAbiolinks: an R/Bioconductor package for integrative analysis of TCGA data. Nucleic Acids Res (2016) 44(8):e71. 26704973 10.1093/nar/gkv1507 PMC4856967

[20] Mayakonda A Lin DC Assenov Y Plass C Koeffler HP. Maftools: efficient and comprehensive analysis of somatic variants in cancer. Genome Res (2018) 28(11):1747–56. 10.1101/gr.239244.118 PMC6211645 30341162

[21] Yoshihara K Shahmoradgoli M Martínez E Vegesna R Kim H Torres-Garcia W. Inferring tumour purity and stromal and immune cell admixture from expression data. Nat Commun (2013) 4:2612. doi: 10.1038/ncomms3612 24113773 PMC3826632

[22] Chen B Khodadoust MS Liu CL Newman AM Alizadeh AA. Profiling tumor infiltrating immune cells with CIBERSORT. Methods Mol Biol (Clifton NJ). (2018) 1711:243–59. doi: 10.1007/978-1-4939-7493-1_12 PMC5895181 29344893

[23] Yang W Soares J Greninger P Edelman EJ Lightfoot H Forbes S. Genomics of Drug Sensitivity in Cancer (GDSC): a resource for therapeutic biomarker discovery in cancer cells. Nucleic Acids Res (2013) 41(Database issue):D955–61. 10.1093/nar/gks1111 PMC3531057 23180760

[24] Jiang P Gu S Pan D Fu J Sahu A Hu X. Signatures of T cell dysfunction and exclusion predict cancer immunotherapy response. Nat Med (2018) 24(10):1550–8. doi: 10.1038/s41591-018-0136-1 PMC6487502 30127393

[25] Charoentong P Finotello F Angelova M Mayer C Efremova M Rieder D. Pan-cancer immunogenomic analyses reveal genotype-immunophenotype relationships and predictors of response to checkpoint blockade. Cell Rep (2017) 18(1):248–62. doi: 10.1016/j.celrep.2016.12.019 28052254

[26] Balar AV Galsky MD Rosenberg JE Powles T Petrylak DP Bellmunt J. Atezolizumab as first-line treatment in cisplatin-ineligible patients with locally advanced and metastatic urothelial carcinoma: a single-arm, multicentre, phase 2 trial. Lancet (London England). (2017) 389(10064):67–76. doi: 10.1016/S0140-6736(16)32455-2 27939400 PMC5568632

[27] Kim ST Cristescu R Bass AJ Kim KM Odegaard JI Kim K. Comprehensive molecular characterization of clinical responses to PD-1 inhibition in metastatic gastric cancer. Nat Med (2018) 24(9):1449–58. doi: 10.1038/s41591-018-0101-z 30013197

[28] Miao D Margolis CA Gao W Voss MH Li W Martini DJ. Genomic correlates of response to immune checkpoint therapies in clear cell renal cell carcinoma. Sci (New York NY). (2018) 359(6377):801–6. doi: 10.1126/science.aan5951 PMC6035749 29301960

[29] Kato S Goodman A Walavalkar V Barkauskas DA Sharabi A Kurzrock R. Hyperprogressors after immunotherapy: analysis of genomic alterations associated with accelerated growth rate. Clin Cancer Res (2017) 23(15):4242–50. doi: 10.1158/1078-0432.CCR-16-3133 PMC5647162 28351930

[30] Remon J Passiglia F Ahn MJ Barlesi F Forde PM Garon EB. Immune checkpoint inhibitors in thoracic Malignancies: review of the existing evidence by an IASLC expert panel and recommendations. J Thorac Oncol (2020) 15(6):914–47. doi: 10.1016/j.jtho.2020.03.006 32179179

[31] Ritchie ME Phipson B Wu D Hu Y Law CW Shi W. limma powers differential expression analyses for RNA-sequencing and microarray studies. Nucleic Acids Res (2015) 43(7):e47. doi: 10.1093/nar/gkv007 25605792 PMC4402510

[32] Picard E Verschoor CP Ma GW Pawelec G. Relationships between immune landscapes, genetic subtypes and responses to immunotherapy in colorectal cancer. Front Immunol (2020) 11:369. doi: 10.3389/fimmu.2020.00369 32210966 PMC7068608

[33] Galon J Bruni D. Approaches to treat immune hot, altered and cold tumours with combination immunotherapies. Nat Rev Drug discovery. (2019) 18(3):197–218. doi: 10.1038/s41573-018-0007-y 30610226

[34] Punekar SR Weber JS. Intratumoral therapy to make a “Cold” Tumor “Hot”: the jury is still out. Clin Cancer Res (2022) 28(23):5007–9. doi: 10.1158/1078-0432.CCR-22-2427 36161479

[35] Dong L Lyu X Faleti OD He ML. The special stemness functions of Tbx3 in stem cells and cancer development. Semin Cancer Biol (2019) 57:105–10. doi: 10.1016/j.semcancer.2018.09.010 30268432

[36] Billen LP Shamas-Din A Andrews DW. Bid: a bax-like BH3 protein. Oncogene (2008) 27 Suppl 1:S93–104. 19641510 10.1038/onc.2009.47

[37] Chu A Zirngibl RA Manolson MF. The V-ATPase a3 subunit: structure, function and therapeutic potential of an essential biomolecule in osteoclastic bone resorption. Int J Mol Sci (2021) 22(13). 10.3390/ijms22136934 PMC8269383 34203247

[38] Liu XJ Zhao HC Hou SJ Zhang HJ Cheng L Yuan S. Recent development of multi-target VEGFR-2 inhibitors for the cancer therapy. Bioorganic Chem (2023) 133:106425. 10.1016/j.bioorg.2023.106425 36801788

[39] de Souza JG Starobinas N Ibañez OCM. Unknown/enigmatic functions of extracellular ASC. Immunology (2021) 163(4):377–88. 10.1111/imm.13375 PMC8274145 34042182

[40] Chen X Kang R Kroemer G Tang D. Broadening horizons: the role of ferroptosis in cancer. Nat Rev Clin Oncol (2021) 18(5):280–96. 10.1038/s41571-020-00462-0 33514910

[41] Lin CF Lin CM Lee KY Wu SY Feng PH Chen KY. Escape from IFN-γ-dependent immunosurveillance in tumorigenesis. J Biomed science. (2017) 24(1):10. 10.1186/s12929-017-0317-0 PMC5286687 28143527

[42] Zhang K Zhu S Li J Jiang T Feng L Pei J. Targeting autophagy using small-molecule compounds to improve potential therapy of Parkinson’s disease. Acta Pharm Sin B (2021) 11(10):3015–34. doi: 10.1016/j.apsb.2021.02.016 PMC8546670 34729301

