# Supplementary figures and images for: Leveraging diverse cell-death related signature predicts the prognosis and immunotherapy response in renal clear cell carcinoma
Source: Front Immunol. 2023 Dec 11;14:1293729. doi: 10.3389/fimmu.2023.1293729 (PMC10749459; doi:10.3389/fimmu.2023.1293729)

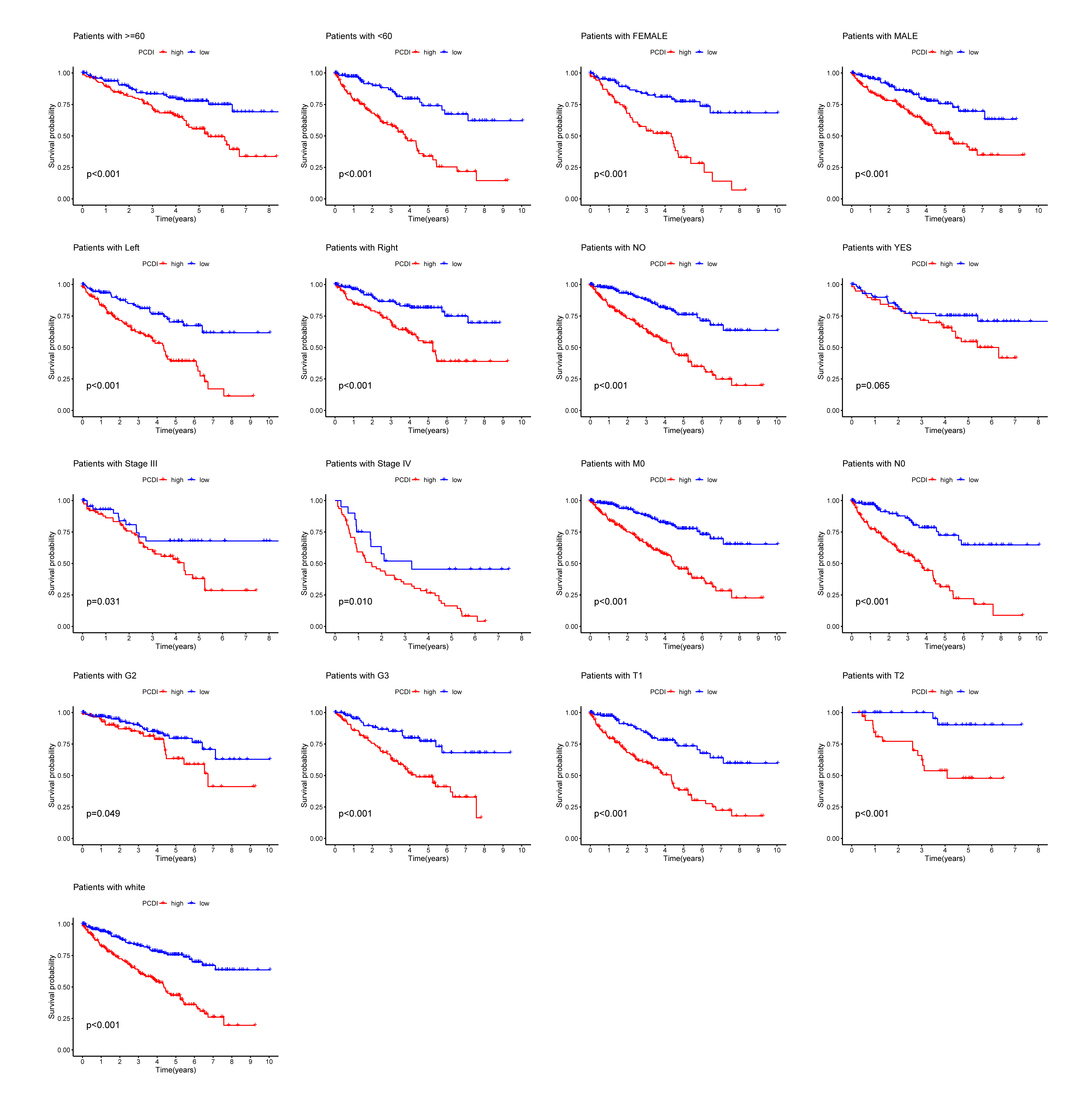

Supplement: Supplementary Figure 1 — Prognosis curve among clinical subgroups. [file Image_1.tif]

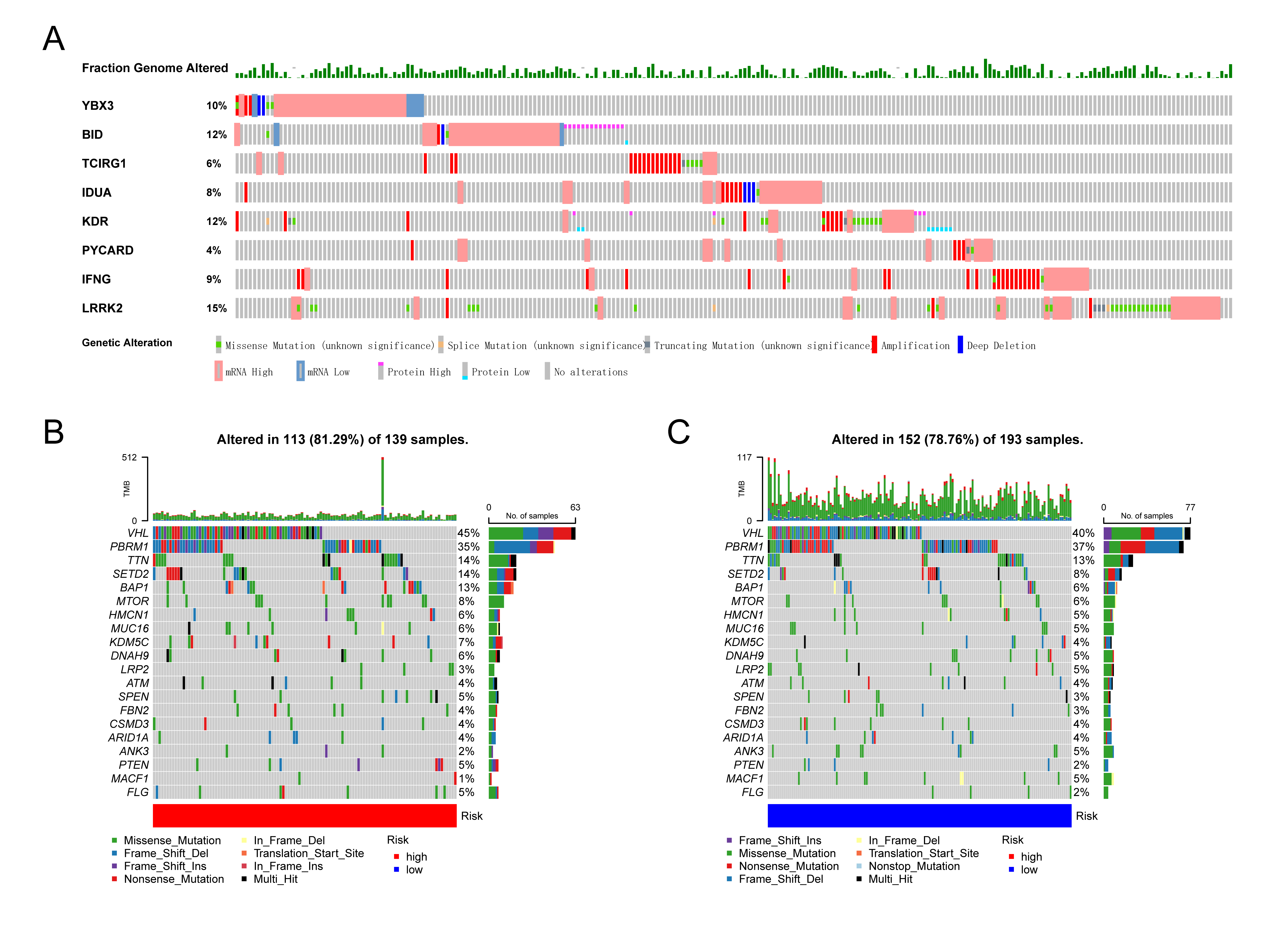

Supplement: Supplementary Figure 2 — Association of PCDI with somatic mutations. (A) Mutation rates of the eight genes for which PCDI was constructed. Waterfall plots of somatic mutations in the high PCDI group (B) and low PCDI group (C). [file Image_2.tif]

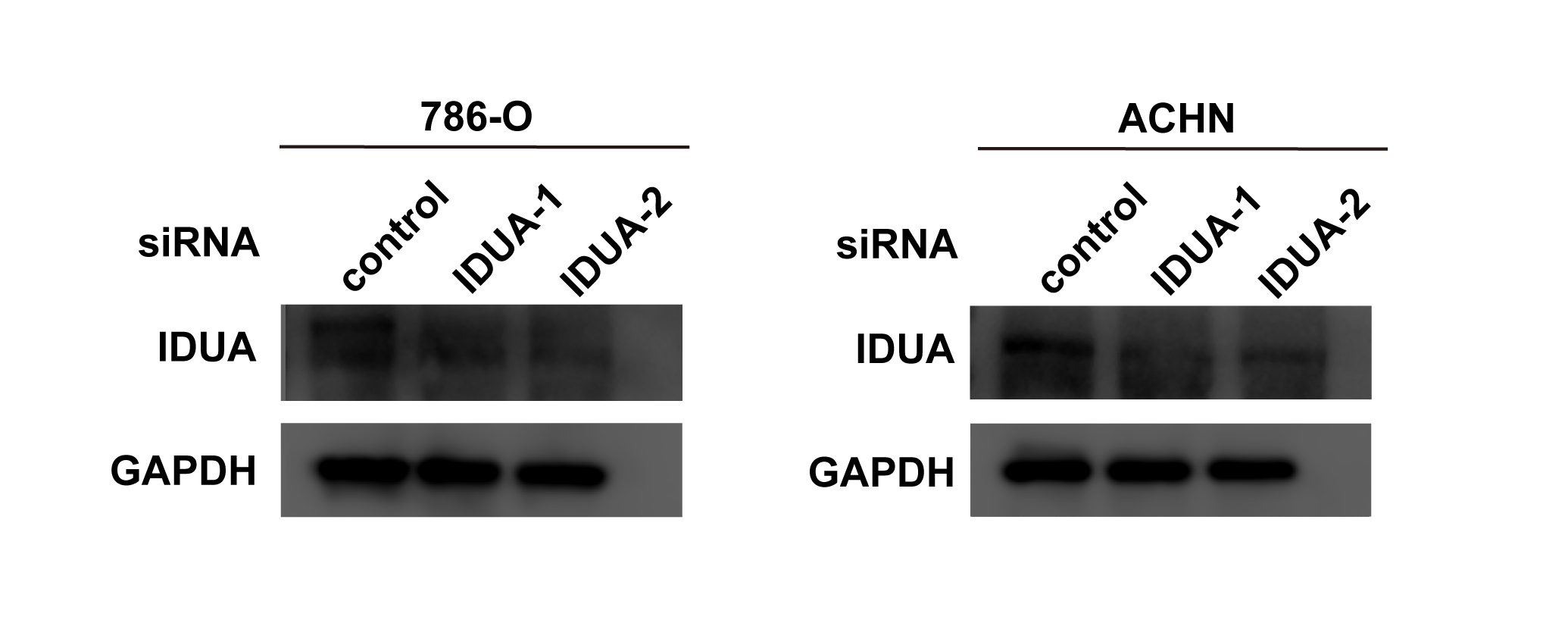

Supplement: Supplementary Figure 3 — Western blotting for validating the knock-down effect of IDUA gene in RCC cell lines 786-O and ACHN. [file Image_3.tif]
